# Characterization and Function of Circular RNAs in Plants

**DOI:** 10.3389/fmolb.2020.00091

**Published:** 2020-05-19

**Authors:** Peijing Zhang, Sida Li, Ming Chen

**Affiliations:** ^1^Department of Bioinformatics, State Key Laboratory of Plant Physiology and Biochemistry, College of Life Sciences, Zhejiang University, Hangzhou, China; ^2^James D. Watson Institute of Genome Sciences, Zhejiang University, Hangzhou, China

**Keywords:** circRNA, plant, circularization, characterization, regulation, stress response, bioinformatics

## Abstract

CircRNAs are covalently closed-loop single-stranded RNA molecules ubiquitously expressing in eukaryotes. As an important member of the endogenous ncRNA family, circRNAs are associated with diverse biological processes and can regulate transcription, modulate alternative splicing, and interact with miRNAs or proteins. Compared to abundant advances in animals, studies of circRNAs in plants are rapidly emerging. The databases and analysis tools for plant circRNAs are constantly being developed. Large numbers of circRNAs have been identified and characterized in plants and proved to play regulatory roles in plant growth, development, and stress responses. Here, we review the biogenesis, characteristics, bioinformatics resources, and biological functions of plant circRNAs, and summarize the distinct circularization features and differentially expression patterns comparison with animal-related results.

## Introduction

Circular RNAs (circRNAs) are a unique group of endogenous non-coding RNAs (ncRNAs) for their distinct closed-loop structures. CircRNAs are formed in the non-canonical “back-splicing” event, in which the 5′ and 3′ ends are attached by covalent bond (Jeck et al., 2013; Memczak et al., 2013; Jeck and Sharpless, 2014). Different from linear RNAs, circRNAs form closed-loop structures by covalent bond linking the 5′ and 3′ ends. Therefore, this type of ncRNAs is more stable than linear RNAs and not easily degraded by Ribonuclease R (RNase R) (Suzuki et al., 2006). Although circRNAs have been observed for decades, they are typically considered as by-products of aberrant RNA splicing (Nigro et al., 1991; Cocquerelle et al., 1993). In the recent decade, numerous circRNAs have been identified and annotated in diverse species as high-throughput sequencing technologies and bioinformatics tools advance. The published research exhibited that circRNAs are ubiquitous and abundant in all eukaryotes, such as mammals, worms, insects, fish, plants, fungi, and protists, and even in prokaryotic archaea (Danan et al., 2012; Jeck et al., 2013; Memczak et al., 2013; Wang et al., 2014; Westholm et al., 2014; Ivanov et al., 2015; Ye et al., 2015).

The majority of circRNAs are generated from protein-coding genes and consist of a single exon or multiple exons (Guo et al., 2014). Despite lack of 5′ caps and poly(A) tails, circRNAs produced in the nucleus are normally transported to the cytoplasm (Salzman et al., 2012), while a few circRNAs that are generated from excised intron lariats preferentially localize to the nucleus (Zhang et al., 2013). Although back-splicing is generally less efficient than linear splicing and their expression level are relatively low, circRNAs can accumulate in specific cell types or tissues in a temporally regulated manner (Rybak-Wolf et al., 2015; Veno et al., 2015) owing to their high stability (Jeck et al., 2013; Memczak et al., 2013). These results suggest the potential functions of circRNAs. Emerging evidence has shown that circRNAs are involved in the regulation of gene expression at transcriptional and post-transcriptional levels. They are reported to function as miRNA sponges (Hansen et al., 2013) or RNA-binding proteins (RBP) sponges (Ashwal-Fluss et al., 2014) and are ideal biomarkers (Lukiw, 2013). It is disclosed in the latest research that proteins and peptides could derive from circRNAs comprising internal ribosomal entry sites (IRESs) (Pamudurti et al., 2017; Yang et al., 2017).

The characterization of circRNAs in plants is comparatively less than the comprehensive systematic analysis in animals. Thousands of circRNAs have been identified in nearly 30 plant species, including the model plants, crops, and Chinese herbal medicines (Ye et al., 2015; Wang et al., 2016; Zuo et al., 2016; Dong et al., 2019). Plant circRNA information is available in various databases, such as PlantCircNet (Zhang et al., 2017) and PlantcircBase (Chu et al., 2018), which collect more than 200,000 circRNAs from different research groups in total. Studies have shown that there are several differences in the circularization mechanisms of circRNAs in plants and animals. Flanking intronic complementary sequences of circularized exons have been demonstrated to be significantly important for circRNA biogenesis in animals (Zhang et al., 2014). Nonetheless, enrichment of repetitive elements or reverse complementary sequences do not appear to be found in flanking sequences of identified plant circRNAs (Lu et al., 2015; Ye et al., 2015). Thus, the circularization of plant circRNAs may be regulated by alternative mechanisms that are yet to be found.

In this review, we outline the recent advances in the biogenesis and functions of circRNAs in plants. The distinct features and circularization mechanisms of plant circRNAs are summarized by comparing them with related studies in animals. Then available bioinformatics resources, expression patterns and diverse functions of plant circRNAs are described. Particularly, the putative roles of plant circRNAs in responses to biotic and abiotic stresses are well summarized in **Table 2**.

## Biogenesis and Discovering of Plant CircRNAs

**Figure 1:**
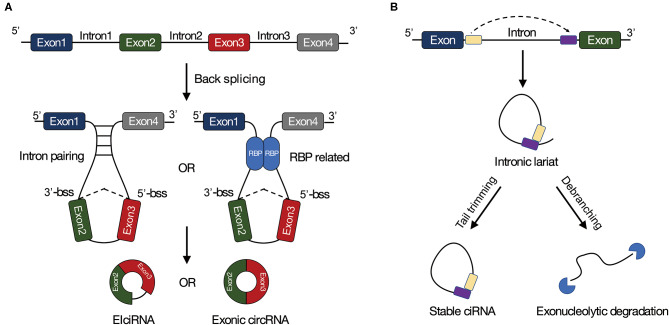
Biogenesis of circRNAs. **(A)** Long flanking introns, inverted repeat elements, and RNA binding proteins are facilitated to back-splicing. **(B)** Circular intronic RNAs (ciRNAs) can be generated from intronic lariat precursors that escape from debranching. Bss, back-splice site; EIciRNA, exon-intron circRNA; ciRNA, circular intronic RNA.

Although the mechanisms of circRNA biogenesis are not very clear, two well-known models have been proposed for circRNA formation. CircRNAs are usually circularized from canonical splicing sites (Jeck et al., 2013; Memczak et al., 2013), which means back-splicing requires canonical splicing machinery (Ashwal-Fluss et al., 2014; Starke et al., 2015). Moreover, back-splicing is normally mediated by base pairing between inverted repeat elements locating in upstream and downstream introns (Zhang et al., 2014; Ivanov et al., 2015), or by dimerization of RBPs that combine with specific motifs in the flanking introns (Figure 1A) (Conn et al., 2015). CircRNAs are also generated from lariat precursors during exon-skipping or from intron lariats that escape debranching (Figure 1B) (Kelly et al., 2015).

rRNA-depleted total RNA-seq is widely used in early genome-wide profiling studies of circRNAs. Previous studies have exploited this type of publicly available datasets for circRNA identification and characterization in plants (Ye et al., 2015). Considering circular structures and relatively low expression levels of circRNAs, an additional protocol that using linear RNase R for library preparation has become the preferred method for greatly enriching circRNAs. This method has been widely used in plant circRNA studies and yielded high confidence circRNAs in maize, soybean, bamboo, tomato, and grape (Zuo et al., 2016; Chen L. F. et al., 2018; Gao et al., 2019; Luo et al., 2019; Wang Y. S. et al., 2019).

As mentioned, circRNAs are created through back-splicing where the downstream 3′ donor splice sites are joined to the upstream 5′ acceptor splice sites (Figure 1A). Two terminal sequences flanking the back-splice sites constitute the sequencing reads crossing back-splice junctions, which is normally ignored by mapping algorithms. These features have been exploited newly in developed bioinformatics tools to specifically detect circRNAs. These tools differ in the strategy of identifying circRNAs, so that they could be assigned to two categories (Chen et al., 2015). The first one is pseudo-reference-based approach, which relies entirely on the accurate genome annotation with all alternatively spliced mRNA isoforms, such as KNIFE (Szabo et al., 2016) and NCLscan (Chuang et al., 2016). Prior to reading mapping, all possible circRNA sequences are reconstructed based on genome annotation by shuffling exon-exon junctions (Szabo et al., 2016). The second group termed fragment-based approach, identify back-splice junctions from mapping information of multiple split alignments, including MapSplice (Wang et al., 2010), find_circ (Memczak et al., 2013), and CIRI (Gao et al., 2015). This approach has been extensively used in plant circRNA research, which infers all possible back-splicing events based on reads mapping information rather than the prior knowledge of gene annotation.

The locus-specific profiling of circRNAs that using genomic positions of back-splice sites, can be adapted for validating, quantitating, and investigating previously characterized circRNAs. For example, reverse transcription and PCR (RT-PCR) is more capable for circRNAs validation than northern blotting (Kristensen et al., 2019), droplet digital PCR (ddPCR) and reverse transcription quantitative PCR (RT-qPCR) are both available for circRNAs quantitation (Maheshwari et al., 2017; Li et al., 2018). However, genome-wide profiling and locus-specific methods could detect unique back-splice sites in circRNAs, while they cannot determine internal splicing patterns of circRNA formation.

## Characterization of Plant CircRNAs

### Circularization

CircRNAs can arise from a wide range of genomic positions and combinations, including exons, introns, or intergenic regions. The biogenesis of circRNAs basically depends on back-splicing of pre-mRNAs, which is conserved in eukaryotes. Significant enrichment of reverse complementary sequences comprising short repeat elements can be detected in flanking introns of circularized exons in animals, which are essential for circRNA circularization (Ashwal-Fluss et al., 2014; Liang and Wilusz, 2014). For example, circularized exons are bracketed by long introns comprising Arthrobacter luteus (Alu) elements in humans (Jeck et al., 2013). In addition, RNA secondary structures and RNA-binding proteins are associated with circRNA biogenesis, and the sequence length of the flanking introns of circularized exons significantly alter circularization efficiency (Conn et al., 2015; Kramer et al., 2015). Unlike animal circRNAs, comparatively few plant circRNAs contain reverse and repetitive complementary sequences in the intronic sequences flanking exons (Lu et al., 2015; Ye et al., 2015; Zhao T. et al., 2017; Zhu et al., 2019), suggesting that intron-pairing-driven circularization may not be the primary mechanism for plant circRNA biogenesis. It has been proved that transposons and their reverse-complement counterparts enrich in maize circRNA flanking regions, showing their affection to circRNA biogenesis (Chen L. et al., 2018).

CircRNAs are generated from back-splicing which covalently link spliced down-stream splice donors with upstream splice acceptors. In the main spliceosome for splicing of most introns of eukaryotes, GT and AG terminal dinucleotides locate in the 5′ and 3′ end, respectively (Szczesniak et al., 2013). However, the mechanisms for spliceosomes selection and splicing signals of circularization are poorly characterized. In plants, circRNAs have been shown to harbor non-GT/AG splicing signals, while the majority exonic circRNAs are spliced by canonical GT/AG signal in animals (Ye et al., 2017). In Arabidopsis, grape and cotton, the majority of identified circRNAs are spliced by canonical splicing signal (Sun et al., 2016; Zhao T. et al., 2017; Gao et al., 2019), which is disagreement with the results in rice. Hundreds of circRNAs are shared with non-canonical splicing signals in rice (Ye et al., 2017), such as GC/CG, CT/GC, and GC/GT, as well as in cucumber (Zhu et al., 2019) and chloroplast of *A. thaliana* (Liu et al., 2019). Interestingly, the circRNA identification methods (find_circ and CIRI2) only consider canonical GT/AG splicing in cucumber and chloroplast of *A. thaliana*. Recent studies have revealed that back-splice sites of circRNAs are flexible and the alternative splicing of circRNAs is prevalent and much of the alternative splicing of circRNAs occurred nearby canonical splicing sites (Starke et al., 2015; Chen et al., 2016; Szabo et al., 2016). Thus, the splice signal patterns of circRNA circularization need to be verified in more plant species.

### Properties and Conservation

CircRNAs are derived from all the chromosomes, as well as mitochondrial and chloroplast genomes in plants. The length of circRNAs ranges from <200 bp to longer than 100 kb, normally <1 kb. In plants, circRNAs are mainly between 200 and 600 bp, only a few of them >2 kb (Ye et al., 2017). Consistent with animals, most plant circRNAs are generated from exons of a single gene, some of them from introns, intergenic regions, untranslated regions (UTRs) or more than one gene (Ye et al., 2015). The results of gene structure annotations demonstrated that most host genes prefer to produce one circRNA than produce more than one circRNA, and few of circRNAs are generated from different loci of the same gene. CircRNA isoforms can be derived from the same locus through alternative circularization. The expression of circRNAs are much lower than their linear counterparts and correlates with the expression of their parental gene transcripts. Though, the internal regulatory mechanism of these correlations needs further research. For example, the expression of Ac_ciRNA_04842 and its derived gene *Achn372061* is positively correlated in kiwifruit (Wang et al., 2017); in rice, overexpressing Os08circ16564 significantly inhibits the expression of its parental gene AK064900 (Lu et al., 2015). CircRNAs are highly conserved in different plant species. More than 700 orthologous genes pairs that produce circRNAs were found between Arabidopsis and rice (Ye et al., 2015). Another study of eight plant species has shown that circRNA host orthologous genes accounting for nearly 20% of genes that produce circRNAs (Zhu et al., 2019).

### Bioinformatics Resources for Plant CircRNAs

High-throughput sequencing technology enables millions of circRNA sequencing reads to be accumulated in a short time period. To deal with the large number of RNA-seq datasets and have deeper understanding of circRNA biogenesis, new algorithms for efficient and accurate identification of circRNA are constantly being developed, including find_circ (Memczak et al., 2013), CIRCexplorer (Zhang et al., 2014), KNIFE (Szabo et al., 2015), and CIRI (Gao et al., 2015). However, these tools have differential performance in terms of sensitivity, accuracy, and computational costs when detecting circRNAs from RNA-Seq datasets. Comparative analyses of circRNA identification tools revealed that CIRI, KNIFE, and CIRCexplorer had better performance in terms of balancing precision and sensitivity (Zeng et al., 2017) and combining different tools could achieve more reliable predictions (Hansen et al., 2016). These comparison results provide useful guidance for improving algorithms and using current tools by researchers. PcircRNA_finder is developed specifically for plant circRNA detection, which combined five different tools to provide a more comprehensive, precise, and sensitive prediction method (Table 1) (Chen et al., 2016). CircPro is developed for investigating the protein-coding ability of circRNAs, which is an automated analysis pipeline that integrates five tools (Table 1) (Meng et al., 2017).

**Table 1:** An overview of bioinformatics resources for plant circRNAs.

**Name**	**Description**	**Web links**	**Latest release**	**Reference**
PcircRNA_finder	An integrated software for circRNA prediction in plants.	http://ibi.zju.edu.cn/bioinplant/tools/manual.htm	2017	Chen et al., 2016
CircPro	An integrated tool for circRNA protein-coding potential.	http://bis.zju.edu.cn/CircPro/	2017	Meng et al., 2017
AtCircDB	A tissue-specific database for Arabidopsis circular RNAs.	http://genome.sdau.edu.cn/circRNA	2018	Ye et al., 2019
PlantCircNet	A database of plant circRNA-miRNA-gene regulatory networks.	http://bis.zju.edu.cn/plantcircnet/	2018	Zhang et al., 2017
ASmiR	A comprehensive database of miRNA targets in alternatively spliced linear and circRNAs.	http://forestry.fafu.edu.cn/bioinfor/db/ASmiR	2019	Wang H. Y. et al., 2019
CropCircDB	A database for crops in response to abiotic stress.	http://deepbiology.cn/crop/	2019	Wang K. et al., 2019
PlantcircBase	A comprehensive database of plant circRNAs in 16 organisms.	http://ibi.zju.edu.cn/plantcircbase/index.php	2019	Chu et al., 2017, 2018
CircFunBase	A database for functional circular RNAs.	http://bis.zju.edu.cn/CircFunBase/index.php	2019	Meng et al., 2019

Increasing numbers of circRNA datasets have been generated and exploded in plants, several databases have been established to effectively organize and manage these datasets (Table 1). Comparing with animals, the plant circRNA databases are relatively insufficient in datasets and types. PlantcircBase (Chu et al., 2017, 2018) and PlantCircNet (Zhang et al., 2017) are comprehensive resources for plant circRNAs, containing published and new identified circRNAs information from different plant species, genome browsing, and putative mRNA-miRNA-circRNA interaction networks in corresponding species. Besides, PlantcircBase provides structure visualization of specific circRNA and validation information by PCR and sequencing. AtCircDB (Ye et al., 2019) is developed for analyzing the tissue specificity of circRNAs in Arabidopsis, while circRNA information in crops response to abiotic stress is summarized in CropCircDB (Wang K. et al., 2019). Though only two crops, maize and rice, are collected in the database. CircFunBase (Meng et al., 2019) provides circRNA information with experimentally validated and computationally predicted functions and visualized circRNA-miRNA interaction networks. ASmiR (Wang H. Y. et al., 2019) contains alternative splicing information form linear and circular RNAs in plants and their interaction information with miRNAs. However, plant species, information of phylogenetic conservation, cell-type, tissue or development stage expression, functional annotation, and interaction with other molecules are still insufficient in various databases, which will greatly promote circRNA research in plants.

## Function of Plant CircRNAs

### CircRNAs Act as miRNA Sponges

**Figure 2:**
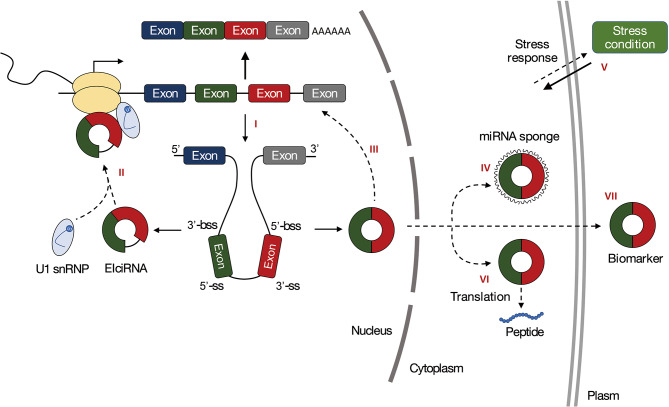
Functions of circRNAs in plants. **(I)** The processing of circRNAs can affect the splicing of their linear counterparts. **(II)** CircRNAs can regulate transcription of their parental genes. **(III)** CircRNAs can regulate the splicing of their linear cognates. **(IV)** CircRNAs can act as miRNA sponges. **(V)** CircRNAs can regulate gene expression in response to biotic or abiotic stresses. **(VI)** CircRNAs can be translated. **(VII)** CircRNAs are promising biomarkers.

Due to previous studies in animals, the most striking function of circRNAs is to act as miRNA sponges or participate in miRNA-related pathways to regulate gene expression (Figure 2IV). The transcripts that contain multiple miRNA-binding sites and inhibit miRNA activity are called miRNA sponges (Ebert et al., 2007), also called competing endogenous RNAs (ceRNAs) in animals or target mimicry in plants (Franco-Zorrilla et al., 2007). These transcripts could be used to explore the function of miRNAs. Due to their loop structures, circRNAs are resistant to RNA exonucleases. These high stabilities make circRNAs have an advantage as miRNA sponges, with half-lives more than 48 h, while the half-lives of their linear counterparts are <20 h (Jeck et al., 2013). The circRNA CiRS-7 is a canonical miRNA sponge playing roles in inhibiting miR-7. In humans, ciRS-7 (also known as CDR1as) contains more than 70 conserved miR-7 binding sites and can strongly suppress miR-7 activity, resulting in increased levels of miR-7 targets (Hansen et al., 2013). Some studies have shown that circRNAs are putative miRNA sponges in plants, but few direct experimental evidence has been proposed. Moreover, compared with animals, plant circRNAs acting as miRNA sponges account for a smaller proportion of the total circRNAs and have lesser miRNA-binding sites (Ye et al., 2015; Zuo et al., 2016).

It is reported that only 6.6 and 5.0% of circRNAs contained putative miRNA-binding sites in rice and Arabidopsis, respectively (Ye et al., 2015). Another study in rice has been shown that 31 circRNAs harbors two or more putative miRNA-binding sites, while in total 235 circRNAs have miRNA-binding sites (Lu et al., 2015). Besides, 53 sea buckthorn circRNAs, 30 cucumber circRNAs, 25 chinese cabbage circRNAs, and 9 pepper circRNAs are predicted to act as miRNA sponges (Wang H. Y. et al., 2019; Zhang G. Y. et al., 2019; Zhu et al., 2019; Zuo et al., 2019), which required further experimental validation. In *Arabidopsis thaliana*, five circRNAs derived from flowers may function as miRNA sponges while one of them has been experimentally validated (Frydrych Capelari et al., 2019). Based on the hypothesis that circRNAs and mRNAs are targeted by the same miRNA, the putative ceRNA networks have been investigated in Arabidopsis leaves, which suggests the regulatory roles of circRNAs in leaf senescence base on differential expression patterns of mRNAs, circRNAs, and miRNAs (Meng et al., 2018). By interacting with miRNA, circRNAs may play regulatory roles in a variety of processes, including metabolic processes, developmental processes, reproductive processes, abiotic, and bionic stress response. Nevertheless, their authenticity requires further experimental validation and crosslinking immunoprecipitation and high-throughput sequencing (CLIP-seq) can help for it (Chi et al., 2009).

### CircRNAs in Stress Response

Previous studies have demonstrated that plant circRNAs exhibit specific cell-type, tissue, or developmental stage expression patterns and circRNAs expression is usually induced under various environmental stresses (Figure 2V; Table 2), including drought, chilling, heat, nutrient deficiency, or pathogen invasion. These suggest that circRNAs may like other ncRNAs, such as miRNAs and lncRNAs, which are crucial to plant growth and development, as well as biotic or abiotic stresses response. Evidence has shown that those differentially expressed circRNAs could regulate plant in responses to stresses by interacting with miRNAs and regulating the expression of stimulus-responsive genes. The regulatory roles and potential functions of circRNAs could be inferred and experimentally verified by establishing the circRNA-mediated ceRNA network under stress conditions.

**Table 2:** Studies of circRNAs in plant stress responses.

	**Plant Stress**	**Plant Species**	**Tissues**	**Number of Differentially Expressed circRNAs**	**Year**	**Reference**
Biotic	Pseudomonas syringae pv. *actinidiae* infection	Kiwifruit	Root/Leaf	584	2017	Wang et al., 2017
	TYLCV infection	Tomato	Leaf	115	2018	Wang J. Y. et al., 2018
	MIMV-Infected	Maize	Leaf	160	2018	Ghorbani et al., 2018
	Verticillium wilt	Cotton	Root/Stem	280	2018	Xiang et al., 2018
Abiotic	Nutrient Depletion	*Oryza sativa* L., *Arabidopsis thaliana*	Root	27	2015	Ye et al., 2015
	Cold	Tomato	Fruit	163	2016	Zuo et al., 2016
	Dehydration	Wheat (*Triticum aestivum* L.)	Leaf	62	2017	Wang et al., 2016
	Low-nitrogen	Wheat (Triticum aestivum L.)	Root	6	2018	Ren et al., 2018
	Drought	Birch-leaf pear (*Pyrus betulifolia* Bunge)	Leaf	33	2018	Wang J. et al., 2018
	Chilling	Bell peppers (*Capsicum annuum* L. cv. Jingtian)	Fruit	36	2018	Zuo et al., 2018
	Heat	Cucumber (*Cucumis sativus* L.)	Leaf	6	2018	He et al., 2019
	Heat	*Arabidopsis thaliana*	Seedling	1583	2018	Pan et al., 2018
	Heat	Radish	Leaf	3	2019	Yang et al., 2019
	Copper	Citrus	Root/Leaf	45/17	2019	Fu et al., 2019
	Drought	Arabidopsis (*Arabidopsis thaliana*), maize (*Zea mays*)	Leaf	1843/1283	2019	Zhang P. et al., 2019
	Cold	Grape (*Vitis vinifera*)	Leaf	475	2019	Gao et al., 2019
	Salt	Cucumber (*Cucumis sativus*)	Root/Leaf	1934/44	2019	Zhu et al., 2019
	Calcium	Chinese cabbage (*Brassica rapa* L. ssp. pekinensis)	Leaf	616	2019	Wang W. H. et al., 2019
	Low-Phosphorus Stress	Soybean	Root	120	2020	Lv et al., 2020

CircRNAs were firstly identified under the biotic stress condition was in Arabidopsis leaves under pathogenic interaction (Sun et al., 2016). In kiwifruit, circRNAs were also identified differentially expressed under pathogen invasion (Wang et al., 2017). In total, 584 circRNAs have been shown differential expression patterns during *Pseudomonas syringae* pv. *actinidiae* (Psa) infection and their expression are related to the stage of infection (Wang et al., 2017). Besides, a list of circRNAs related to plant defense response have been identified by network analysis (Wang et al., 2017). Later studies indicate that circRNAs can function as negative regulators of tomato yellow leaf curl virus (TYLCV) interaction in tomato (Wang J. Y. et al., 2018), play regulatory roles in the Verticillium wilt response in cotton (Xiang et al., 2018) and are response to maize Iranian mosaic virus (MIMV) infection in maize (Ghorbani et al., 2018).

CircRNAs have been shown to be differentially expressed under abiotic stresses, including nutrient depletion, high light, heat, chilling, drought, or salt. However, the regulatory mechanism and specific biological significance of plant circRNAs during these conditions remain to be elucidated. In the samples from Oryza sativa roots with phosphate-starvation condition and *A. thaliana* leaves with light treatment, circRNAs were firstly identified in plants, and 27 circRNAs in rice were found to display stress-specific expression patterns under phosphate deficiency condition, of which 6 were up-regulated and 21 were down-regulated (Ye et al., 2015). These results indicated the potential roles of plant circRNAs in response to stress, furthermore, the stress-specific expression patterns are also found in other plant species with different biotic stress conditions. Analyses have shown that 36 and 163 differentially expressed circRNAs were identified in chilled bell pepper (Zuo et al., 2018) and chilled tomato fruit (Zuo et al., 2016) respectively, 475 differentially expressed circRNAs were identified in grape leaves under cold stress (Gao et al., 2019). The grape Vv-circATS1, derived from *glycerol-3-P acyltransferase*, has been found to improve cold tolerance in *Arabidopsis* by regulating the expression of stimulus-responsive genes, such as *CSD2, PRXCA, PME41, LOX3*, and *WRKY48*. Under dehydration-stressed conditions, differentially expressed circRNAs have been detected in wheat (Wang et al., 2016), pear (Wang J. et al., 2018), maize, and Arabidopsis (Zhang P. et al., 2019). Moreover, the similar patterns of expressional changes were observed in crops under nutrient depletion (Darbani et al., 2016; Wang W. H. et al., 2019; Lv et al., 2020), metal ion toxicity (Fu et al., 2019) or salt (Zhu et al., 2019). In addition, researchers found that the stress condition could alter lengths of circRNAs, numbers of circularized exons and alternative circularization events in Arabidopsis (Pan et al., 2018).

### CircRNAs Regulate Gene Expression

Accumulating evidence has proved that circRNAs are involved in the regulation of gene expression. The processing of circRNAs can affect the splicing of their linear counterparts (Figure 2I), and regulate the transcription of their parental genes (Figure 2II). In human, exon-intron circRNAs (EIciRNAs) enhance transcription of circRNA host genes through association with U1 snRNP. EIciRNAs have been identified in plants and implicated in gene regulation in biotic stress response (Zhao W. et al., 2017). In Arabidopsis, CircSEP3 that derived from SEPALLATA3 (SEP3) gene has been shown to regulate transcription and splicing of their linear counterparts (Figure 2III). CircSEP3 can bind strongly to its cognate DNA locus and form an RNA:DNA hybrid, whereas the linear RNA with the same sequence bind to the DNA much more weakly. The circRNA:DNA formation would result in transcriptional pausing and leading to the formation of alternatively spliced SEP3 mRNA with exon skipping (Conn et al., 2017). These together suggest that circRNAs can modulate gene expression at both transcription and splicing levels. In addition, the correlations between circRNAs and their parental genes have been indicated in studies of plant circRNAs, the mechanistic basis of these correlations needs further experimental validation.

### CircRNAs Can Be Translated

Due to the lack of key components for the canonical cap-dependent translation, such as 5′ caps or poly(A) tails, circRNAs are considered untranslatable. However, recent research in mammals revealed that circRNAs translation could be driven by IRESs (Abe et al., 2015) and promoted by N6-Methyladenosine (m6A) RNA modification (Meyer et al., 2015; Yang et al., 2017) (Figure 2VI). Although a number of circRNAs have been predicted to contain putative open reading frames (ORFs) with IRESs, a few circRNAs have proven to act as protein templates, and functions of peptides derived from circRNAs remain to be explored (Legnini et al., 2017; Pamudurti et al., 2017; Zhang et al., 2018). For example, peptides FBXW-185aa that are translated from circ-FBXW7 may inhibit tumorigenesis of brain cancer (Yang et al., 2018). FBXW-185aa has been shown to interact with the deubiquitinating enzyme USP28, protecting USP28 from binding to a key regulator of tumorigenesis, FBXW7α. Therefore, FBXW-185aa prevents FBXW7α-induced degradation by antagonizing USP28-induced c-Myc stabilization. Moreover, circRNAs that contain large ORFs and m6A-modified sites in junction sequences have been detected to encode hundreds of peptides (Tang et al., 2020). For example, m6A-modified circE7 could be translated to produce E7 oncoprotein, which is directly responsible for HPV-induced carcinogenesis (Zhao et al., 2019). The latest evidence also shows that m6A modified circRNA could inhibit innate immunity (Chen et al., 2019). However, there is no circRNA in plants has been reported to be translated. The studies of m6A modification in Arabidopsis have been reported (Zhou et al., 2017), translatable circRNAs and their biological functions would be proposed in plants as research progresses.

### CircRNAs Act as Biomarkers

It has been illustrated by previous research that circRNAs are universal in diverse cell types, conserved in different species and exhibit specific expression patterns, which makes them potential biomarkers (Figure 2VII). Moreover, circRNAs have been shown to participate in diverse physiological and pathophysiological processes, including cancers (Li et al., 2015; Qin et al., 2016; Zhu et al., 2017), cardiovascular disease (Satoh et al., 2013), neurological disease (Grapp et al., 2013) and diabetes (Zhao Z. et al., 2017), suggesting that circRNAs are becoming the emerging biomarkers for diagnosis and treatment of human diseases. CircRNAs are also regarded as aging biomarkers in *Drosophila* research (Westholm et al., 2014). Biomarkers have been investigated and used in breeding applications for a couple of years in plants (Yang et al., 2011). Due to their characteristics, circRNAs are emerging biomarkers in plants. In Arabidopsis, circRNAs have been shown to act as bona fide biomarkers of alternative splicing variants (Conn et al., 2017). The study in maize and Arabidopsis suggests that circRNAs play essential roles in plant drought response, and can be used as effective biomarkers in genetic improvement of crop drought tolerance (Zhang P. et al., 2019). Hence, the biomarker is an interesting research topic in plant circRNAs.

## Conclusions and Future Perspectives

With large numbers of circRNAs being identified, more insights have been given into circRNAs, which has become a promising research hotspot. CircRNAs usually exhibit specific expression patterns and are also induced by stress conditions, suggesting that circRNAs may be a new regulatory factor at the transcriptional and post-transcriptional levels. Evidence indicates that circRNAs participate in diverse biological processes, they can act as miRNA sponges, interact with many different RBPs, be translated into peptides or act as promising biomarkers. Compared with related studies in animals, the research of plant circRNAs are relatively less, and lack of experimental and functional verification. The formation mechanism of plant circRNAs is based on bioinformatics analysis rather than convincing experimental evidence, which is still in the theoretical stage. The characterization and function of plant circRNA have been proposed and verified, but there are still quite a few prediction results, especially the interactions between circRNAs and miRNAs. In addition, the coding ability of circRNAs and their individual function roles in plant growth and developments, such as leaf senescence, flower development, fruit maturation, and response to biotic and abiotic stresses, have not been investigated so far, which may account for primary research topics of circRNAs in plants. We still need more observation and validated results about the biogenesis mechanism, specific regulatory roles, and functions of these molecules, as well as large-scale quantification, full-length sequencing and functional verification of circRNAs. In addition, the differences between plant and animal circRNAs will also be an interesting topic.

## Author Contributions

PZ and MC wrote and revised the manuscript. SL prepared the figures and revised the manuscript. All authors read and approved the final manuscript.

## Conflict of Interest

The authors declare that the research was conducted in the absence of any commercial or financial relationships that could be construed as a potential conflict of interest.
